# Female Reproductive Tract Organ-on-Chips: Modeling Barrier Function and Drug Transport

**DOI:** 10.3390/pharmaceutics18030280

**Published:** 2026-02-24

**Authors:** Shiqing Zhou, Zizhao Xu, Jie Shen

**Affiliations:** Department of Pharmaceutical Sciences, School of Pharmacy and Pharmaceutical Sciences, Northeastern University, Boston, MA 02115, USA; zhou.shiq@northeastern.edu (S.Z.); zi.xu@northeastern.edu (Z.X.)

**Keywords:** female reproductive tract, organ-on-chip, drug permeation, drug transport, women’s health, barrier function

## Abstract

Female reproductive tract (FRT) disorders such as maternal conditions and gynecological cancers represent a significant global health burden. However, women’s health, and particularly locally acting therapies targeting the FRT, has historically been underprioritized in drug development and translational research. Developing safe and effective therapies requires a clear understanding of drug transport across FRT barriers. Conventional in vitro culture systems and animal studies fail to recapitulate the physiological complexity of the human FRT, including stratified mucosal architecture, functional mucus barriers, microbiome interactions, as well as dynamic hormonal regulation. Recently, organ-on-chip (OoC) microfluidic platforms, integrating human cells with precisely controlled perfusion, have emerged as advanced in vitro systems capable of recreating dynamic physiological microenvironments. This review summarizes the major anatomical and physiological barriers of the FRT, including the vagina, cervix, endometrium, and placenta, and discusses critical design considerations for the development of FRT-on-chip models. We highlight the advanced OoC developed to study infection, drug permeation, hormonal responses, and maternal–fetal interface dynamics. Finally, future perspectives are outlined, including the integration of immune components, vascularization strategies, and multi-organ systems to better simulate inter-organ communication. Collectively, these advances underscore the potential of FRT-on-chip models as predictive platforms for preclinical drug screening, toxicity evaluation, and personalized medicine.

## 1. Introduction

Female reproductive tract (FRT) disorders represent a substantial global health burden, accounting for millions of deaths and significant disability worldwide, yet remain disproportionately under-represented in drug development and translation research [1]. Among these, maternal disorders contribute approximately 290,000 deaths annually [2], representing the leading cause of FRT-related mortality worldwide [3,4]. Cervical cancer alone claims over 260,000 lives each year, predominantly affecting those in resource-limited settings where access to prevention, screening, and early detection remains inadequate [5]. Beyond mortality, chronic conditions such as endometriosis affect an estimated 190 million women globally, causing debilitating pelvic pain, infertility, and markedly reduced quality of life [6,7]. Despite this significant disease burden, women’s health research remains critically underfunded, with only approximately 5% of global research and development funding allocated to women’s health research (4% for women’s cancers and 1% for all other women-specific conditions) [8]. This stands in stark contrast with the fact that women comprise approximately half of the global population and spend 25% more time in poor health than men [9]. Collectively, these conditions underscore the urgent need for improved research models and therapeutic strategies focused on reproductive and women’s health research.

The FRT, encompassing the vagina, cervix, endometrium, and placenta, functions as a series of dynamic biological barriers. These tissues undergo menstrual cycle-dependent epithelial remodeling, hormone-regulated mucus secretion, and cyclic alternations in permeability, which collectively regulate hormone signaling, maintain microbial homeostasis, and control the transport of nutrients and therapeutic agents [10,11]. Barrier-specific mechanisms further govern molecular transport: epithelial tight junction proteins (e.g., claudins, occludins, ZO-1) regulate paracellular permeability in the vaginal and endometrial epithelia [10,11]; the cervicovaginal mucus layer creates a size- and charge-selective diffusion barrier influencing nanoparticle penetration and drug residence time; and the placental syncytiotrophoblast, enriched in ATP-binding cassette efflux transporters (P-gp, BCRP, MRPs), restricts fetal xenobiotic exposure while facilitating nutrient exchange [12,13]. Intravaginal administration offers opportunities for localized and targeted therapy, particularly in contraception and infection management [14]. However, effective FRT drug delivery remains challenging due to the unique physiological characteristics of the vaginal environment, including rapid drug clearance driven by vaginal self-cleaning mechanisms, acidic pH, hormonally regulated changes in mucus viscosity and endometrial permeability, enzymatic activity from epithelial and microbial sources, and a *Lactobacillus*-dominated microbiome that produces lactic acid and metabolic enzymes and [15,16,17,18]. Together, these physiological factors influence drug stability, retention, and transport kinetics, making a mechanistic understanding of drug transport across FRT barriers essential for optimizing female-specific administration routes such as intravaginal and intrauterine delivery and for ensuring maternal–fetal safety.

Early in vitro FRT models primarily relied on two-dimensional (2D) monolayer cultures due to their affordability and experimental simplicity. Representative examples include the vaginal epithelial cultures, endometrial stromal cultures [19], and maternal–fetal interface cell systems developed to study placenta drug transport [20,21]. However, 2D culture systems fail to reproduce key aspects of native tissue architecture and functions, since cells maintained in two dimensions often lose physiological morphology, polarity, and critical cell–cell and cell–matrix interactions necessary for accurate barrier function and differentiation [22,23,24]. More fundamentally, the FRT’s barrier properties are hormone-dependent: vaginal epithelial stratification, cervical mucus composition, endometrial permeability, and placental transporter expression all undergo dynamic changes in response to fluctuating estradiol and progesterone levels across the menstrual cycle and pregnancy [25,26]. Static 2D cultures fail to recapitulate the complex cyclical hormonal regulation and inter-organ communication that define the hormone-responsive FRT, making them inherently limited for modeling FRT drug transport [27]. Animal models, although widely used, also present inherent limitations for the study of women’s health, such as species-specific differences in anatomy, hormone regulation, and reproductive cycling that hinder translational relevance [28]. For example, the human menstrual cycle spans approximately 28 days, whereas the rodent estrous cycle lasts only 4–5 days and exhibits markedly different hormone fluctuation patterns [29,30,31]. Because key determinants of barrier permeability, including epithelial stratification, mucus secretion, and efflux transporter expression, are hormonally regulated and cycle-dependent in humans, interspecies differences contribute to poor predictive accuracy for intravaginal drug formulations and hormone therapies. Drug absorption and residence time observed in rodent models frequently fail to correlate with clinical outcomes [32,33]. Furthermore, ethical and regulatory constraints restrict the use of animals, particularly in pregnancy-related studies [34].

Organ-on-chip (OoC) technologies have emerged as advanced bioengineering platforms designed to overcome many of the limitations associated with conventional in vitro and animal models. OoC systems are microfabricated 3D cell culture devices that recapitulate key structural, biochemical, and functional features of human tissues and organs in vitro [35]. By integrating microfluidics and precisely controlled microscale fluid flow [36], OoC platforms enable the reconstruction of the dynamic mechanical and biochemical environments absent in traditional static culture systems, thereby more accurately simulating cellular microenvironments and organ-level functions [27]. For instance, vagina-on-chip and cervix-on-chip have been developed to recreate the stratified epithelium and mucus barrier of the lower FRT, enabling studies on the microbial balance and infection processes [37,38,39]. Endometrium-on-chip models incorporate stromal and vascular components to model hormone-dependent epithelial barrier function and have been used to evaluate the effect of contraceptive drugs on endometrial vascular permeability and blood vessel regression [40,41]. Among FRT-related OoC platforms, placenta-on-chip models are the most extensively studied and have successfully reconstructed the maternal–fetal interface, enabling a mechanistic investigation of nutrient exchange, drug transport, and nanoparticle toxicity [42,43,44]. More recently, multi-organ OoC systems have been introduced to integrate hormonal communication across the ovary, fallopian tube, uterus, cervix, and liver, allowing for the partial recapitulation of endocrine cycles and more comprehensive assessment of drug responses within the female reproductive system [27,45].

Despite these advances, the application of OoC technologies to women’s reproductive health research remains at an early developmental stage, with substantial opportunities to further enhance physiological relevance and expand their use in evaluating drug transport and safety across the FRT barriers. As the limitations of traditional in vitro and animal models become increasingly apparent, OoC platforms offer a human-relevant approach to study reproductive barrier function, hormonal regulation, and therapeutic transport. The present review fills this gap by systematically examining OoC models across the FRT, with specific emphasis on their design considerations, quantitative transport studies, drug evaluation, and safety assessment. We summarize the current advances in OoC models of the vagina, cervix, endometrium, and placenta, highlighting their unique contributions in modeling barrier biology and evaluating drug delivery systems. Emerging multi-organ FRT-on-chip platforms and their potential to model systemic hormone communication are also discussed. Taken together, these OoC technologies offer a promising framework for advancing translational therapeutic research and addressing persistent gaps in women’s reproductive health.

## 2. Female Reproductive Tract Barriers

To effectively design OoC platforms that recapitulate these complex physiological functions, a comprehensive understanding of FRT anatomy, regional barrier architectures, and immunological specialization is essential, as these features directly inform the structural, cellular, and functional requirements of physiologically relevant in vitro models. The FRT is anatomically divided into two regions: the upper tract, which includes the ovaries, fallopian tubes, and uterus, and the lower region, comprising the cervix, vagina, and vulva [46]. The ovaries are responsible for oocyte production and storage, as well as the synthesis of hormones [47]. The fallopian tubes extend from the ovaries to the uterus, where they capture ovulated oocytes and facilitate their transport to the uterine cavity [48]. The uterus is a hollow, muscular organ that supports embryo implantation and fetal development [49]. In the lower FRT, the cervix forms the interface between the uterus and the vagina, which connects the reproductive tract to the external environment [50]. Immune regulation within the FRT is both anatomically and functionally specialized. The lower FRT, which is continuously exposed to the external environment, is predominately governed by innate immune defenses, whereas the upper FRT supports immune modulation to permit sperm survival, embryo implantation, and fetal tolerance [51]. Key immune cell populations within the FRT include T cells, macrophages, dendritic cells, and neutrophils. Although less abundant, B cells contribute to mucosal immunity through the production of secretory IgA (sIgA) and IgG [52]. These immune cells play critical roles in maintaining barrier integrity through immune surveillance and regulated cytokine signaling. However, dysregulated inflammatory responses can disrupt epithelial tight junctions, leading to increased permeability and altered pathogen susceptibility and drug transport across FRT barriers [53,54].

**Figure 1:**
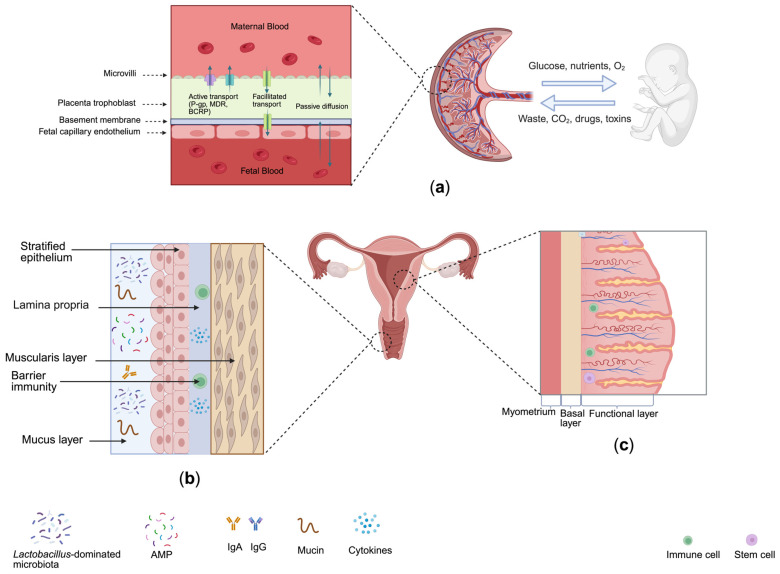
Overview of key physiological barriers in the female reproductive tract and their structural characteristics: (**a**) Placental (maternal–fetal) barrier: A multilayered structure composed of the placenta trophoblast, basement membrane, and fetal endothelium, mediating bidirectional exchange of nutrients, oxygen, and waste. Key transport mechanisms include passive diffusion, facilitated transport, and active efflux via transporters such as P-gp, MDR, and BCRP. (**b**) Vaginal mucosal barrier: A stratified squamous epithelium overlaid by a mucus layer enriched in mucins, antimicrobial peptides (AMPs), and immunoglobulins (IgA, IgG); (**c**) Endometrial (epithelial–stromal) barrier: A columnar epithelium layer supported by a fibroblast-rich stroma compartment, exhibiting hormone-dependent changes in permeability during the menstrual cycle; Note: The barrier diagrams are arranged anatomically.

Collectively, these anatomical and immunological features form a multi-layered barrier system throughout the FRT (Figure 1). Together, these interconnected barriers define physiological features, highlighting the need for advanced in vitro platforms capable of replicating region-specific structure, immune regulation, and permeability.

### 2.1. Vaginal Mucosal Barrier

In the lower FRT, the vaginal mucosal barrier, composed of stratified squamous epithelium, cervicovaginal mucus, and a *Lactobacillus*-dominated microbiota, serves as the primary defense against pathogens while regulating drug absorption [55,56].

#### 2.1.1. Epithelial Structure and Function

The vagina epithelium constitutes the first line of defense within the lower female reproductive tract. Anatomically, the vagina is a distensible, muscular canal, approximately 7–10 cm in length. The vagina wall is composed of three layers: an outer fibrous adventitia, a middle layer of smooth muscle, and an inner mucosa layer [57]. The mucosa is lined with stratified squamous epithelium cells that form a robust, multilayered surface with characteristic folds (rugae), thereby providing a large surface area for absorption [58]. However, the stratified architecture also imposes diffusional resistance, particularly limiting the permeation of macromolecules and hydrophilic compounds due to multiple cell layers and the lipid-enriched intercellular matrix [39,59]. Beyond serving as a physical barrier, vaginal epithelial cells actively participate in innate immune defense by secreting antimicrobial peptides (AMPs) and cytokines, forming a responsive chemical shield that is rapidly activated upon exposure to pathogens or irritants [60]. Vaginal epithelial cells also facilitate the transport of immunoglobulins, including IgA and IgG into the reproductive tract lumen, where they function in concert with the resident microbiota against bacterial and fungal pathogens and other harmful agents.

Vaginal epithelial permeability is dynamically regulated by reproductive processes such as sperm transport, capacitation, fertilization, and implantation [61]. Selective permeability is regulated by multiple ion channels, including epithelial sodium channel (ENaC) and cystic fibrosis transmembrane conductance regulator (CFTR), which together fine-tune luminal fluid composition and barrier function [62].

#### 2.1.2. Microbiota and pH Maintenance

A defining feature of the vaginal epithelium is its close interaction with the resident microbiota. The vaginal microbiome plays a critical role in maintaining reproductive tract health and contributes significantly to barrier integrity and protection against infection. *Lactobacillus* species, which dominate the vaginal microbiota (approximately 70–80%), competitively inhibit pathogen colonization and maintain an acidic pH (4–4.5) through production of lactic acid [17]. Disruption of this microbial balance (dysbiosis) can compromise epithelial barrier integrity by inducing inflammation, oxidative stress, and the immune dysregulation [63]. Dysbiosis can also influence drug delivery through: (1) altering vaginal pH, thereby affecting drug solubility and release [64]; (2) changes in microbial enzymatic activity, which impacts drug stability [65]; (3) modifications in mucus rheology, thus altering drug diffusion [66]; and inducing inflammation-mediated transporter upregulation, which may reduce drug absorption [67]. Under these conditions, mucosal barrier function is governed by complex interactions among the vaginal microbiota, host immune system, and hormonal signaling across the reproductive tract [68].

#### 2.1.3. Mucus Layer Composition and Role

Overlaying the vaginal epithelium is the cervicovaginal mucus layer, which serves as a dynamic, semipermeable interface connecting the vagina and cervix. The mucus consists of ~95% water and a network of gel-forming mucins (primarily mucin 5B), along with immunoglobulins, defensins, enzymes, and metabolites secreted by epithelial cells and commensal microorganisms [18,69]. Functionally, cervicovaginal mucus performs both protective and regulatory roles. The viscoelastic mucus layer forms a selective barrier that traps pathogens and limits their adhesion to the epithelial surface, while simultaneously supporting beneficial microbiota, such as *Lactobacilli*, to maintain vaginal health [70]. Foreign particles trapped within the mucus are removed through mucus secretion and flow or eliminated by immune surveillance mechanisms [71]. Hormonal fluctuations across the menstrual cycle further modulate mucus viscosity, pH, and mucin composition, thereby regulating its permeability to sperm, microorganisms, and exogenous compounds [72].

Together, the vaginal epithelium, resident microbiota, and mucus layer form an integrated mucosal barrier system that governs the penetration and transport of drugs, nanoparticles, and biologics within the lower reproductive tract. These characteristics make the vaginal mucosa a critical consideration for the design of effective mucosal drug delivery platforms.

### 2.2. Endometrial Barrier (Epithelial–Stromal Barrier)

The endometrium functions as a dynamic epithelial–stromal barrier that modulates hormone-dependent permeability, supports embryo implantation, and maintains immune tolerance during early pregnancy [73]. Anatomically, the endometrium lines the uterine cavity and is composed of a columnar epithelial layer overlying a multicellular stromal compartment [74]. Structurally, it can also be divided into two distinct zones: the basal layer and the functional layer. The basal layer lies adjacent to the myometrium and remains relatively stable throughout the menstrual cycle, providing structural support and serving as a protective physical barrier against pathogens [75,76]. In contrast, the functional layer, comprising glandular epithelium, blood vessels, and connective tissues, undergoes cyclic remodeling under the regulation of ovarian hormones [77]. Estradiol predominates during the proliferative phase, promoting epithelia and stromal growth, whereas progesterone governs the secretory phase following ovulation, driving differentiation and preparation for implantation [78]. Together, these zones form a hormonally regulated and immunologically active barrier capable of balancing protection against infection with the physiological requirement to accommodate embryo implantation. The cycling remodeling and selective permeability of the endometrial epithelial–stromal interface make it a critical system for studying drug absorption, hormone therapy, and implantation biology. This cyclic remodeling directly influences uterine drug disposition through phase-specific immune and barrier modulation. For instance, progesterone-driven immune tolerance during the secretory phase may enhance drug penetration but increase susceptibility to infection [78,79], whereas heightened immune surveillance during the proliferative phase promotes inflammatory responses that can limit bioavailability [80,81]. Additionally, cycle-dependent changes in vascular permeability and tight junction integrity generate phase-specific absorption patterns [82]. These hormonally regulated dynamics underscore the importance of endometrium-on-chip platforms for evaluating uterine drug transport, therapeutic efficacy, and safety.

### 2.3. Placenta Barrier (Maternal–Fetal Barrier)

At the maternal–fetal interface, the placental barrier governs nutrient exchange and regulates drug transfer between the maternal and fetal circulations [83]. Functionally, the highly selective blood–placenta barrier enables bidirectional transport of substances such as gases (oxygen and carbon dioxide), nutrients, and metabolic waste while restricting the passage of potentially harmful xenobiotics [84]. Structurally, the placental barrier is formed primarily by syncytiotrophoblast cells, which constitute the principal interface separating maternal blood from fetal blood [85]. The multinucleated trophoblast layer is characterized by dense microvilli on the maternal-facing surface, increasing the surface area for nutrients (e.g., glucose, amino acids, lipids), oxygen and metabolic waste exchange [86]. Beneath the syncytiotrophoblast lies fetal connective tissue that supports the fetal capillary network, with the innermost layer formed by the fetal capillary endothelium, which mediates final transfer of substances into the fetal circulation [87,88].

What distinguishes the placental barrier from other FRT barriers is its exceptionally high expression of protective ATP (adenosine triphosphate)-binding cassette (ABC) efflux transporters that actively restrict fetal exposure to xenobiotics. P-glycoprotein (P-gp/ABCB1) and breast cancer resistance protein (BCRP/ABCG2) are highly expressed in the syncytiotrophoblast and actively pump substrates such as chemotherapeutics (e.g., paclitaxel), and antidiabetic agents (e.g., glyburide) back into the maternal circulation [87,89]. While small, lipophilic compounds such as caffeine can readily cross the placenta via passive diffusion, and drugs structurally similar to endogenous substrates may utilize carrier-mediated transport pathways, the robust efflux transporter expression provides critical fetal protection against maternal drug exposure [12,13]. Collectively, these transport mechanisms define the placenta’s unique selectivity profile and are critical determinants of maternal–fetal drug disposition.

Owing to its multilayered structure and complex transport physiology, the placental barrier represents one of the most sophisticated selective barriers in human biology. Its tightly regulated permeability makes it a focal point for modeling drug transfer, nanotoxicology, and maternal–fetal pharmacokinetics (PK), particularly within placenta-on-chip platforms.

### 2.4. Comparative Transport Mechanisms Across FRT Barriers

Despite structural and anatomical differences, drug transport across FRT barriers shares common transport mechanisms that govern drug permeation and therapeutic delivery, operating primarily through passive diffusion, facilitated diffusion, and active transport. The relative contribution of each mechanism is determined by molecular properties such as size, lipophilicity, charge, and affinity for tissue-specific transporters [90,91].

Passive diffusion is the predominant mechanism for small (<500 Da), lipophilic molecules across all FRT barriers and is driven by concentration gradients across the epithelial membranes [92]. Facilitated diffusion involves carrier-mediated transport of compounds structurally similar to endogenous substrates, such as amino acids or glucose analogs, and does not require cellular energy input [93]. In contrast, active transport relies on ATP-dependent pumps to move substrates against concentration gradients and includes both influx transporters (e.g., organic anion transporting polypeptides, OATPs) [94] and efflux transporters [91]. Among these, ABC efflux transporters, particularly P-gp/ABCB1, multidrug resistance-associated proteins (MRPs), and BCRP/ABCG2, are widely expressed throughout the FRT and serve as critical protective mechanisms against xenobiotic exposure [95,96]. Transporter expression and regulation vary across FRT regions. In the vagina and cervix, P-gp is expressed in stratified squamous epithelium, albeit at lower levels than in the placenta, and contributes to limiting drug absorption while protecting against xenobiotics [90,97]. In the endometrium, P-gp expression is hormonally regulated, with increased expression during the progesterone-dominant secretory phase, potentially influencing drug delivery to the uterine cavity [26,33]. The placenta exhibits the highest expression of efflux transporters among FRT tissues, reflecting its critical role in restricting fetal exposure to therapeutic agents and environmental toxins [98].

Together, these shared transport mechanisms, in combination with tissue-specific barrier architecture and transporter expression profiles, determine drug distribution, residence time, and therapeutic efficacy across the FRT (Table 1). Understanding these comparative transport processes is essential for the rational design of OoC platforms that accurately recapitulate in vivo drug disposition and for the development of targeted drug delivery strategies tailored to specific anatomical regions.

## 3. OoC Designs for Modeling Drug Transport Across FRT Barriers

This review follows the anatomical sequence encountered by intravaginally administered therapeutics to systematically evaluate OoC systems in the context of drug delivery and transport across FRT barriers. It is also organized to reflect the increasing maturity of OoC applications, moving from early-stage lower-tract models to more application-focused upper-tract platforms. Following intravaginal administration, drug formulations must first undergo dissolution within the vaginal environment. The dissolved drug or nanoparticle-encapsulated drug then encounters the vaginal mucus layer and epithelium, followed by the cervical mucus barrier, which collectively regulate drug residence time, particle diffusion, and ultimately, transport toward the upper reproductive tract. Beyond these lower-tract barriers, effective drug delivery to upper-tract or systemic targets requires transport across the endometrial epithelial–stromal interface, and, during pregnancy, the placental maternal–fetal barrier. Although the FRT acts as a continuous and interconnected barrier system, the maturity and pharmaceutical relevance of existing OoC models vary considerably across anatomical regions (Table 2). Lower-FRT platforms (e.g., vagina-, cervix-on-chip) have demonstrated significant progress in modeling epithelial differentiation, mucus secretion, and host–microbiome interactions; however, quantitative drug permeability and transport studies remain limited due to the complexity of the local microenvironment. In contrast, upper-tract OoC platforms, especially placenta-on-chip models, have achieved greater integration of quantitative transport analyses relevant to drug safety and disposition. This framework highlights current capabilities while highlighting critical gaps that must be addressed to enable predictive drug-testing applications in women’s health.

### 3.1. OoC Models in the Lower FRT

**Figure 2:**
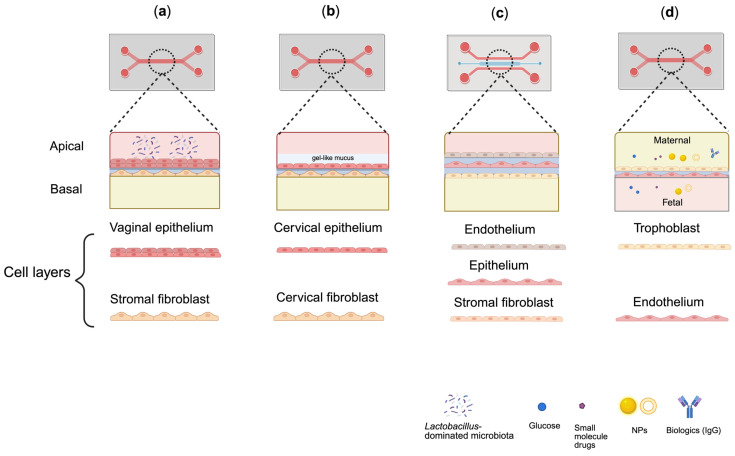
Comparative schematic of reproductive organ-on-chip (OoC) models representing key barriers of the female reproductive tract. Representative OoC platforms recapitulate the structural and functional complexity of FRT barriers through microfluidic co-culture systems. The vagina-on-chip (**a**) comprises apical vaginal epithelial and basal stromal fibroblast layers, supporting *Lactobacillus*-dominated microbiota and hormone-responsive barrier formation. The cervix-on-chip (**b**) features apical cervical epithelial and basal fibroblast layers, with functional mucus secretion that mimics native cervical gel-like mucus and provides microbial barrier function. The endometrium-on-chip (**c**) integrates endothelial, epithelial, and stromal layers that recapitulate hormone-dependent permeability changes. The placenta-on-chip (**d**) consists of trophoblast and endothelial layers perfused through separate maternal and fetal microchannels, enabling investigation of nutrient, small molecule, nanoparticle (NP), and biologic (e.g., IgG) transport across the maternal–fetal barrier.

Recent advances in OoC engineering have enabled the development of first-generation of microphysiological models of the lower FRT (Figure 2). Most vagina- and cervix-on-chip systems are primarily based on commercially available dual-channel microfluidic platforms, such as Emulate CHIP-S1™ [110,111], which support epithelial–stromal co-culture, barrier formation, and microbiome–host interaction studies under dynamic flow. In contrast, dedicated mucus-on-chip platforms that recapitulate cervicovaginal mucus in a reproductive context remain limited. Instead, mucus has either been harvested from cervix-on-chip devices and introduced into vagina-on-chip systems to study inter-tissue crosstalk, or modeled using acellular mucus-on-chip constructs containing reconstituted or synthetic mucin gels, which are primarily intended for permeability screening [112,113,114].

#### 3.1.1. Vagina-on-Chip

As the primary site of topical and mucosal drug delivery, vagina-on-chip platforms provide a physiologically relevant system for evaluating epithelial barrier function, microbiome interactions, and drug permeation. The first reported vagina-on-chip design was capable of supporting co-culture of primary vaginal epithelial cells and resident microbiota under controlled microfluidic conditions [105]. This two-channel microfluidic device (Emulate CHIP-S1™), separated by a porous polymethylsiloxane (PDMS) membrane (7 μm pore size), is incubated with collagen IV (30 μg/mL) and collagen I (200 μg/mL) in Dulbecco’s Modified Eagle Medium (DMEM) at 37 °C with 5% CO_2_ for 2–3 h prior to seeding primary vaginal epithelial cells (3 × 10^6^ cells/mL) and fibroblasts (1 × 10^6^ cells/mL) on opposite sides of the porous membrane in the apical channel. Perfusion of Hanks’ Balanced Salt Solution (HBSS) in the apical channel and β-estradiol-containing differentiation medium in the basal channel at 40 μL/h was selected to ensure effective epithelial differentiation and hormone responsiveness [38]. The model successfully maintained stable colonization by *Lactobacillus crispatus*, representing a healthy vaginal microenvironment, as well as dysbiotic consortia containing *Gardnerella vaginalis*, thereby enabling the investigation of host–microbiome crosstalk under dynamic conditions [105]. This study represented a critical milestone in modeling vaginal physiology in vitro; however, quantitative drug transport measurements remain sparse.

#### 3.1.2. Cervix-on-Chip

The cervix and its mucus layer serve as dynamic regulators of vaginal pH, microbial homeostasis, and drug penetration within the lower FRT. Using the same dual-channel architecture as vaginal systems (Emulate CHIP-S1™), primary cervical epithelial (1.5 × 10^6^ cells/mL) and primary cervical fibroblast cells (0.65 × 10^6^ cells/mL) were co-cultured on opposite sides of a porous membrane, representing the apical and basal compartments, respectively. Under a hormone-responsive perfusion regimen, customized HBSS (pH 5.4) was perfused periodically (30 µL/h for 4 h/day) in the apical channel, while the fibroblast medium was continuously perfused (40 µL/h) through the basal channel, enabling sustained hormone-responsive mucus secretion and the reconstruction of a gel-like cervical mucus barrier [112], key regulators of microbial homeostasis and drug penetration. When integrated with a vagina-on-chip system, cervical mucus effluents introduced into the vaginal channel suppressed pro-inflammatory cytokines and reduced dysbiotic microbiota colonization, demonstrating physiologically relevant cervicovaginal crosstalk mediated by mucus transport [37]. This dual-chip configuration highlighted the role of cervical mucus as an active signaling and protective barrier rather than a passive diffusion layer.

### 3.2. Endometrium-on-Chip

The endometrium represents a dynamic interface between the lower and upper FRT and serves as a critical barrier regulating implantation, hormone responsiveness, immune tolerance, and uterine drug transport. Endometrium-on-chip systems extend microphysiological modeling upstream to the uterine environment, where both vascular permeability and endocrine regulation play central roles [115,116]. Most endometrium-on-chip platforms are organized into three functional compartments: (1) an epithelial channel representing the uterine luminal lining, (2) a stromal channel mimicking the connective tissue matrix, and (3) an endothelial channel reconstructing the vascular interface. Simpler two-channel designs have been reported, including co-cultures of human umbilical vein endothelial cells (HUVECs) with endometrial stromal cells [107] or endometrial epithelial–stromal co-cultures alone [106,117]. More advanced three-layer models integrates HUVECs, endometrial epithelial cells (EECs, commonly the Ishikawa line), and endometrial stromal fibroblasts (ESFs), enabling reconstruction of epithelial–stromal–vascular interactions [40]. Notably, one model replaced immortalized Ishikawa cells with primary endometrial epithelial organoids (EEOs), providing a physiologically relevant alternative that preserves tissue-specific signaling and hormone responsiveness [118]. Collectively, these models recreate a vascularized endometrial microenvironment that closely reflects in vivo cellular interactions and barrier function.

Fabrication of endometrium-on-chip systems most commonly relies on PDMS-based microfluidic devices produced by soft lithography, featuring dual- or multi-channel geometries separated by porous membranes (PDMS) to facilitate nutrient diffusion, cell–cell communication, and imaging across the tissue barrier [40]. Some OoC designs employ resin-based microfabrication to engineer porous membranes that enhance epithelial–stromal attachment and long-term culture sustainability, maintaining cell viability and hormone responsiveness for up to four weeks [107]. Extracellular matrix (ECM) coatings typically include collagen I, Matrigel, or fibrin gels, each supporting distinct levels of adhesion, differentiation, and tissue remodeling. More recently, a synthetic polyethylene glycol (PEG)-based hydrogel crosslinked with matrix metalloproteinase (MMP)-labile peptides has been developed to support co-culture of endometrial organoids and stromal cells [119]. This hydrogel dynamically remodels in response to cellular enzymatic activity, enabling sustained tissue reorganization and providing a robust platform for modeling menstrual physiology and endometriotic disease.

Endothelial channels in endometrium-on-chip systems are typically perfused at flow rates of approximately 1 µL/min, corresponding to shear stresses on the order of near 6 × 10^−3^ dyn s cm^−2^ [107,120]. These conditions promote endothelial tight junction formation, vascular stability, and physiologically relevant perfusion. In contrast, epithelial and stromal compartments are often maintained under static conditions to preserve hormone responsiveness and differentiation [107]. In more complex multi-channel vascularized systems, peripheral fibroblast channels sustain paracrine angiogenic signaling, while central channels facilitate epithelial–vascular communication [40].

Quantitative barrier permeability assays in endometrium-on-chip platforms is commonly assessed using fluorescein Isothiocyanate–dextran (FITC–dextran) as a tracer to calculate the apparent P_app_ [121]. A vascularized endometrium-on-chip model developed by Ahn, J. et al. applied this assay to visualize diffusion across the epithelial–endothelial interface at 10 s intervals, establishing physiological permeability values. The same system was subsequently applied to evaluate levonorgestrel, a synthetic progestin widely used in emergency contraception, at concentrations from 10–10,000 ng/mL. Dose-dependent increases in endometrial permeability and regression of microvessels confirm barrier disruption and anti-angiogenic effects at higher levonorgestrel concentrations. These findings validate endometrium-on-chip platforms as a pharmacologically responsive platform for contraceptive and barrier-modulating drug evaluation [40].

Beyond pharmacological agents, endometrium-on-chip models have been used to study the metabolic regulation of endometrial barrier physiology. In a bovine epithelial–stromal co-culture model, both channels were perfused at 1 µL/min for 72 h and subsequently exposed to increasing glucose concentrations (0.5, 5.0, and 50 mM). This exposure induced dose-dependent transcriptomic and proteomic remodeling, with 21 differentially expressed genes identified in epithelial cells and 191 in stromal cells, highlighting the endometrium’s sensitivity to metabolic status. In contrast, varying insulin levels (1–10 ng mL^−1^) produced minimal transcriptional changes, suggesting selective responsiveness to glucose rather than insulin [106,122].

Current endometrium-on-chip systems capture key aspects of endometrial physiology, enabling quantitative assessment of barrier permeability and hormone-dependent drug responses. More recently, an innovative patient-derived endometrium-on-chip has also been developed, integrating a novel Endometrial Receptivity Scoring system that combines molecular profiling of receptivity markers with quantitative angiogenesis assessment to predict implantation potential. This platform enables personalized infertility management and advances targeted therapies in reproductive medicine [41]. While these endometrium-on-chip models have demonstrated clear value for toxicology, contraceptive evaluation, and barrier-modulating drug studies, several challenges remain. These include incomplete incorporation of immune components, limited control over cyclic hormonal dynamics, and inconsistent reporting of flow rate, shear stress, and ECM stiffness across studies, which can hinder reproducibility.

### 3.3. Placenta-on-Chip

Among the FRT OoC models developed, placenta-on-chip systems have been the most extensively applied in drug transport studies. Owing to the well-defined architecture of the maternal–fetal interface and its central role in regulating fetal exposure, these platforms provide a physiologically and pharmacologically relevant model for studying nutrient exchange, xenobiotic transport, and nanoparticle interactions during pregnancy. Over the past decade, a variety of microfluidic platforms have been engineered to enable quantitative evaluation of apparent P_app_, flux, and transporter-mediated efflux for small molecules, biologics, and nanomaterials.

Most placenta-on-chip systems reconstruct the placental barrier using two opposing cellular compartments: a maternal-facing trophoblast layer (apical side) and a fetal capillary endothelial layer (basal side). Devices are commonly fabricated using PDMS-based microfluidic platforms produced by soft lithography [123], or commercially available multi-channel systems such as OrganoPlate^®^ [124] and AngioPlate™384 [125] employed to improve throughput and standardization. On the maternal side, trophoblast cell lines include BeWo [126] or BeWo b30 cells [44], JEG-3 cells [108], HTR8/SVneo trophoblast cell [127], and ACH- trophoblast cells are widely used [128], with BeWo cells being the most common due to their ease of culture and syncytialization capacity. However, as a cancer-derived line, BeWo cells may not fully recapitulate physiological barrier functions of healthy trophoblasts [109]. To overcome this limitation, placenta-on-chip platforms incorporating human induced pluripotent stem cells (hiPSCs)-derived trophoblasts have been explored, offering improved developmental and functional fidelity [129]. Fetal compartments are typically formed using HUVECs [44] or human placental vascular endothelial cells (HPVECs) [21].

Despite shared design principles, substantial variability exists across platforms in extracellular matrix composition, membrane materials, and perfusion strategies. Most devices employ maternal and fetal microchannels separated by porous polycarbonate or polyethylene terephthalate (PET) membranes (pore size typically ranging from 0.4–3 µm) coated with ECM proteins (e.g., fibronectin, collagen-I or -IV, Matrigel, and fibrin hydrogels) [108], which influence trophoblast differentiation, transporter expression, and barrier integrity. Microfluidic perfusion is typically achieved using syringe pumps (e.g., 20–100 µL/h) or gravity-driven rocking platforms (tilt 7°, 8 min intervals), generating a low-shear environment that approximate intervillous blood flow [108,130,131]. Importantly, wall shear stress has been shown to regulate trophoblast fusion, polarization, and transporter expression, with values around 0.1 dyn/cm^−2^ promoting barrier maturation [132]. Barrier formation generally occurs over 2–9 days, under dynamic perfusion conditions [133].

Placenta-on-chip platforms have been widely applied to investigate glucose transfer, drug transport, and the impact of nanoparticles on the placental barrier [134,135]. Glucose transport studies consistently report glucose transfer rates (~22–35%) that closely align with ex vivo human placental perfusion data and outperform conventional Transwell systems, supporting the improved physiological relevance of OoC platforms [130,136]. The maternal-to-fetal transport of glucose occurs primarily via facilitated diffusion mediated by glucose transporters (GLUTs). Beyond nutrients, placenta-on-chip systems have been used to evaluate endocrine-disrupting compounds, revealing localized oxidative stress and inflammatory responses while preserving certain organ-level transport functions [137,138].

Nanotoxicity studies have further demonstrated the utility of these platforms for assessing gestational-stage-dependent uptake and toxicity. Liposomes uptake in the human placenta was enhanced under biomimetic conditions promoting trophoblast syncytialization (e.g., forskolin treatment) and shear stress [139], suggesting dynamic regulation of placental transport mechanisms during pregnancy. In contrast, copper oxide nanoparticles introduced on the maternal side, were found to impair hormone secretion, disrupt glucose transport, and induce inflammatory responses, collectively compromising placental barrier function. These findings suggest a potential mechanism through which nanoparticle exposure could contribute to abnormal fetal development [140].

Placenta-on-chip have also been applied to assess therapeutic drug permeability and transporter-mediated efflux. Studies using the fluorescently labeled paclitaxel demonstrated dose-dependent fetal exposure limited by P-gp-mediated efflux, consistent with clinical observations [125]. Similar transporter-dependent behavior was observed for glyburide, a model BCRP substrate, confirming efflux activity across the placental barrier [21].

Despite these advances, several limitations remain. The reliance on cancer-derived trophoblast lines (e.g., BeWo or JEG-3) limits physiological relevance for long-term and developmental studies. Furthermore, although glucose transport has been extensively examined, placental transport of essential fatty acids and amino acids remains underexplored, which is one of the essential physiological characteristics of the placenta barrier [141]. Addressing these challenges will be critical for advancing placenta-on-chip platforms toward standardized and predictive pharmaceutical testing.

Representative OoC application for drug transport and safety assessment across the FRT, highlighting quantitative transport readouts and translational relevance, as summarized in Table 3.

### 3.4. Mucus-on-Chip Platforms and Their Potential Integration

The mucus layer is a critical component of cervicovaginal defense, influencing microbial colonization, drug diffusion, and formulation residence time. OoC platforms enable the formation of functional mucus barriers under controlled microfluidic conditions, allowing in situ visualization of mucus turnover, pH dynamics, and hormone-induced compositional changes [142]. Although most current mucus-on-chip are not yet integrated with FRT tissues, they have provided important quantitative insight into mucus-mediated drug transport.

One representative mucus-on-chip design employed microfabricated PDMS pillar arrays to confine mucin solutions and establish a stable mucus–aqueous interface. Fluorescence microscopy was used to track the transport of nanoparticle drug carriers (50 nm and 200 nm, PEG- or pectin-coated) through the mucus layer. These studies demonstrated size- and surface chemistry-dependent transport, with 50 nm nanoparticles diffused efficiently, while particles ≥ 200 nm exhibited restricted movement due to steric hindrance. PEG coatings enhanced mucopenetration, whereas pectin coatings promoted mucoadhesion and prolonged residence time. In a complementary approach, a silicon-based micro-membrane chip simulated the mucus barrier under humidified conditions using a porous membrane (0.4 μm) to support mucus inserts. Permeation assays with caffeine, diclofenac sodium, and FITC–dextran revealed selective diffusion behavior: small molecules permeated in a time-dependent manner, whereas large hydrophilic tracers were effectively excluded [143]. Together, these systems validate the utility of mucus-on-chip platforms for probing size-, surface chemistry-, and formulation-dependent transport across cervicovaginal mucus.

These mucus-on-chip designs demonstrate the feasibility of using microfluidic environments to quantify drug transport across cervicovaginal mucus, a dominant barrier for topical formulations and nanoparticle-based drugs. Their cell-free architecture enables straightforward fabrication, making them valuable screening tools prior to advancing into more complex epithelial co-culture systems. However, the absence of epithelial and stromal components limits their ability to fully recapitulate the vagina mucosal barrier. Future development should emphasize integration with cervicovaginal cell layers, incorporation of dynamic mucus renewal, and standardized rheological characterization to more accurately reflect physiological conditions.

### 3.5. Multi-Organ FRT on Chip

Multi-organ-on-chip platforms have been developed to model the FRT barriers as an interconnected system, enabling investigation of inter-organ communication, endocrine regulation, and systemic drug effects. The EVATAR multi-organ-on-chip platform, represents one of the earliest and most comprehensive efforts to emulate the FRT and its endocrine feedback loops in vitro. This microfluidic system integrates the ovary, fallopian tube, uterus, cervix, and liver, enabling dynamic inter-organ communication under controlled flow conditions. Using mouse ovarian tissues and early secondary follicles maintained for 28 days, EVATAR successfully reproduced hormonal oscillations characteristic of the human menstrual cycle, including physiologically relevant estradiol and progesterone profiles associated with follicular and luteal phases [45]. Importantly, EVATAR also incorporated PK modeling of estradiol distribution, highlighting its pharmaceutical relevance for studying drug–hormone interactions, metabolism under cyclic endocrine conditions and pharmacokinetic–pharmacodynamic (PK-PD) relationships. The platform has also been used to evaluate chemotherapeutic toxicity, demonstrating impairment of ovarian function and synergistic effects arising from hepatic metabolism of agents such as paclitaxel and cisplatin [144].

**Figure 3:**
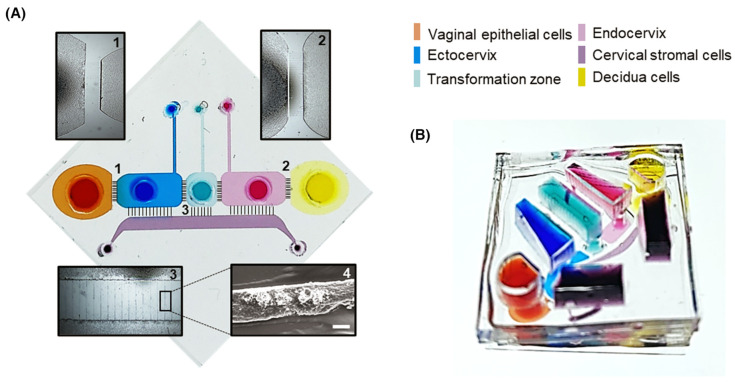
The integrated OoC system connects serially arranged chambers representing vaginal epithelial cells (orange), ectocervix (blue), transformation zone (teal), endocervix (pink), cervical stromal cells (purple) and decidua (yellow) to model ascending infection pathways, with (**A**) showing the schematic design and numbered microscopy images of each tissue compartment, and (**B**) displaying the three-dimensional assembled device, reproduced with permission from Tantengco OAG et al., The FASEB Journal; published by Wiley, 2022 [145].

A six-chamber vagina–cervix–decidua organ-on-chip was developed to investigate host responses to infection by *Ureaplasma parvum*, a common bacterial pathogen associated with preterm birth. The interconnected microchannel architecture enabled co-culture of vaginal, cervical, and decidual tissues in independent media while maintaining tissue interfaces, as illustrated in the multi-channel OoC platform (Figure 3). These studies showed that the *U. parvum* alone did not induce infection unless co-existing with other pathogens [145], underscoring the importance of multi-organ context in infection dynamics. Integration of this system with a feto-maternal interface OoC further enabled assessment of exosome-mediated signaling during pregnancy, revealing that isolated exosomal exposure from infected ectocervical epithelial cells did not trigger preterm birth.

Multi-organ OoC systems highlight the potential of interconnected platforms to model endocrine regulation, infection propagation, and maternal–fetal communication. The progression from single-organ to multi-organ FRT-on-chip systems represents a strategic trade-off between experimental simplicity and physiological complexity, with each approach offering distinct advantages for specific applications. Single-organ platforms (e.g., endometrium-, or placenta-on-chip) are well suited for mechanistic investigation of organ-specific barrier function and enable precise quantitative transport measurements under controlled conditions [103,106]. In contrast, multi-organ systems such as EVATAR capture inter-organ communication and hormonal feedback loops necessary for studying menstrual cycling, infection ascension, and cross-tissue metabolic interactions [45,145]. However, current multi-organ platforms remain experimental research tools rather than validated pharmaceutical testing systems. Key limitations include incomplete incorporation of immune components, simplified hormonal regulation relative to in vivo physiology, challenges in maintaining long-term stability, lack of cross-laboratory standardization, as well as limited validation against clinical data [146,147]. Most multi-organ systems remain academic prototypes requiring substantial technical optimization before adoption for routine drug screening. Nevertheless, proof-of-concept demonstrations of inter-organ signaling and sustained 28-day menstrual cycling [45] highlight their promise for future system-level predictive platforms.

### 3.6. Fabrication Technologies for FRT OoC Platforms

The fabrication of FRT OoC devices employs several microfabrication technologies. PDMS-based soft lithography remains the most widely used approach due to its optical transparency, gas permeability, biocompatibility, and accessible fabrication protocols [37,40,108,123]. However, PDMS’s critical limitation is nonspecific adsorption of hydrophobic drugs, which can underestimate drug concentrations and permeability coefficients [148,149]. Mitigation strategies including surface modification with PEG coatings, albumin blocking, or parylene-C deposition have been explored, though with variable success and added fabrication complexity [150,151,152]. Alternative fabrication approaches utilize thermoplastic materials, including polystyrene, polycarbonate, polymethylmethacrylate (PMMA), and cyclic olefin copolymer (COC), which offer minimal drug adsorption, making them increasingly preferred for quantitative drug permeability studies [153,154,155]. These materials are processed using hot embossing, injection molding, or computer numerical control (CNC) micro-milling techniques with more specialized fabrication equipment [156,157]. Some FRT OoC platforms have successfully employed resin-based photopolymerization to create devices with extended culture capability and improved cell attachment [158,159]. Photocurable resins processed by stereolithography or two-photon polymerization represent emerging approaches that enable complex biomimetic structures, such as placental villous architecture with micrometer resolution [131], though extensive post-processing is required to remove uncured photo-initiators to avoid cytotoxicity [160,161]. The “optimal” fabrication technology depends on the application: PDMS soft lithography remains the most accessible and widely used for early-stage model development, barrier characterization, and studies where optical imaging is essential [162,163]; thermoplastic or surface-modified PDMS devices are preferred when accurate drug concentration measurements and minimal absorption are critical [164].

## 4. Future Perspectives

OoC technology has substantially advanced the modeling of female reproductive barriers, evolving from static monolayer cultures to dynamic, microengineered systems capable of mimicking physiological interfaces under controlled flow and hormonal conditions. In the lower reproductive tract, vagina-, and cervix-on-chip models have successfully modeled stratified epithelium, mucus secretion, and hormone-dependent differentiation. Endometrium-on-chip systems have progressed further by incorporating epithelial, stromal, and endothelial compartments, enabling studies of vascularization, hormone regulation, and barrier permeability. At the maternal–fetal interface, placenta-on-chip platforms have achieved the most extensive application in drug transport research, integrating trophoblast–endothelial bilayers, reproducing transporter expression, and generating maternal–fetal transfer profiles consistent with ex vivo perfusion data. Collectively, these OoC models bridge the gap between conventional in vitro systems and clinical observations, offering powerful tools for evaluating permeability, toxicity, and therapeutic efficacy in women’s health.

Despite these advances, several challenges continue to limit the widespread adoption of reproductive OoC models in pharmaceutical development. Compared with conventional approaches, OoC platforms require specialized microfabrication expertise, incur higher per-sample costs, and involve more complex workflows that increase labor demands and extend study timelines. Reproducibility remains a key concern. Limited commercial harmonization of platforms complicates cross-laboratory reproducibility comparison, and regulatory pathways for OoC data acceptance in drug development are still evolving. In addition, methodological variability represents a significant technical barrier. Fewer than one-third of published studies report critical design and operating parameters such as flow rate, shear stress, ECM stiffness, or membrane pore size, despite their direct influence on barrier integrity and transport behavior. Furthermore, widespread reliance on PDMS complicates drug testing due to nonspecific adsorption of hydrophobic compounds, underscoring the need for alternative materials or functional coating that can avoid hydrophobic drug adsorption. Physiological complexity also remains incomplete. For instance, lower-tract OoC models often lack immune and endothelial components, limiting their ability to capture inflammatory responses and vascular interactions. Cell sourcing presents further challenges, as primary cells offer superior physiological relevance but suffer from donor variability and short lifespans, whereas cancer-derived lines (e.g., BeWo, Ishikawa) lack aberrant barrier properties. Although hiPSC-driven cells and organoids offer promising alternatives, their differentiation and reproducibility require further optimization. Moreover, quantitative validation against human tissue and ex vivo perfusion data is rarely performed, making permeability coefficients and transporter activity difficult to benchmark. Finally, most OoC systems remain low throughput, constraining scalability and integration with automated pharmaceutical screening pipelines.

Future development of reproductive OoC platforms should prioritize cross-organ connectivity to enable assessment of drug transport from the lower to upper FRT. Establishing standardized reporting of design and biomechanical parameters will be essential to enhance reproducibility and facilitate quantitative comparison across studies. Incorporation of immune components, dynamic hormonal regulation, and microbial interactions will be critical for modeling menstrual cycling, infection-related pathologies, and host–microbiome–drug interactions. Furthermore, systemic validation of in-chip permeability and PK outcomes against human ex vivo and clinical datasets will accelerate regulatory confidence and adoption of these models for preclinical drug evaluation.

Reproductive OoC platforms represent a transformative advance in modeling drug transport within the FRT. From microbiome-interactive vagina-on-chip systems to quantitatively validated placenta-on-chip platforms, these technologies provide mechanistic insight into mucosal permeability, hormonal regulation, and maternal–fetal exchange. By providing human-relevant and physiologically controlled models, they overcome species differences that limit animal model predictivity and reduce reliance on scarce ex vivo tissues. They enable mechanistic investigation of reproductive toxicity at the organ level and support safer assessment of drug exposure during pregnancy without clinical risk. Collectively, these advances accelerate development of locally acting intravaginal and intrauterine therapeutics, facilitate personalized medicine approaches, and ultimately improve translational efficiency of FRT drug development while reducing resource-intensive testing and late-stage failure risk.

As these technologies mature and gain regulatory acceptance, they represent a transformative approach to addressing the longstanding therapeutic gaps in women’s reproductive health. Although the field remains in an early developmental stage, the continued convergence of bioengineering, stem cell biology, and modeling is expected to yield integrated, standardized, and patient-derived reproductive OoCs capable of predictive modeling across the female lifespan and pregnancy, narrowing the translational gap between benchtop discovery and clinical therapy.

## Figures and Tables

**Table 1:** Summary of major physiological barriers in the female reproductive tract.

Barrier	Key Structure Features	Functions	Applications
Vaginal mucosa	Stratified squamous epithelium; cervicovaginal mucus layer (~95% (*w*/*w*) water, gel-forming mucin such as MUC5B [99]); *Lactobacillus*-dominated microbiota	Prevents pathogen entry and colonization [18,100]; maintains mucosal integrity and acidic pH [101]	Primary site for topical and mucosa drug delivery studies
Endometrial epithelial–stromal	Columnar epithelial overlying stromal fibroblasts, exhibiting hormonally regulated cyclic remodeling across the menstrual cycle [18]	Regulates barrier permeability; supports embryo implantation [32]; maintains immune tolerance	Models for hormone therapies, drug-induced effects on embryo implantation, and uterine physiology
Placental barrier (maternal–fetal)	Syncytiotrophoblast layer overlying fetal capillary endothelium; transporter-rich exchange interface [102]	Regulates maternal–fetal exchange of gases, nutrients, metabolic waste and xenobiotics (including drugs) [103]; and limits fetal exposure via active efflux mechanisms [104]	Models for maternal–fetal drug safety assessment and nanoparticle transport studies.

**Table 2:** Design parameters and functional summary of OoC models in the FRT.

Model	Chip Architecture	Chip Microenvironment	Cells	Barrier Maturation	Key Strengths	Limitations
Vagina on chip [105]	Two-channel PDMS microfluidic device separated by a 7 µm PDMS porous membrane	Apical channel: intermittent perfusion with HBSS (4 h/day at 40 µL/h); basal channel: continuous perfusion with medium containing β-estradiol at 40 µL/h	Primary human vaginal epithelial cells, uterine fibroblasts	~6 days	Reproduces hormone-responsive squamous epithelium and supports microbiome colonization	Lacks immune components; limited quantitative drug transport data
Cervix on chip [37]	Two-channel PDMS microfluidic device separated by a 7 µm PDMS porous membrane	Apical perfusion with HBSS (4h/day at 30 µL/h) and basal perfusion with medium containing estradiol-17β at 40 µL/h	Primary cervical epithelial cells; primary cervical fibroblasts	~7 days	Mimics hormone-responsive mucus secretion and epithelial–stromal interactions	No endothelial or immune layers; limited quantitative drug transport data
Endometrium on chip [40,106,107]	PDMS dual- or tri-channel systems; ECM coatings (collagen I, fibrin, Matrigel)	Perfusion typically ~1 µL/min; hormone simulation (estradiol and/or progesterone) to mimic proliferative or secretory phases	Endometrial epithelial cells (Ishikawa or primary), stromal fibroblasts, HUVECs	~10–28 days	Recapitulates vasculogenesis, cyclic hormone response, and permeability regulation	Early stage for quantitative drug absorption studies; lacks immune integration
Placenta on chip [43,108,109]	Two-channel PDMS microfluidic device separated by 0.4–3 µm porous membrane; ECM coatings (collagen I/IV, fibronectin)	Maternal channel: perfusion with trophoblast culture medium (10–100 µL/h); fetal channel: perfusion with endothelial growth medium (10–100 µL/h)	BeWo b30, JEG-3, or hiPSC-derived trophoblasts; HUVECs (fetal endothelium)	~3–9 days	Recreates maternal–fetal exchange and transporter-mediated efflux	BeWo limitations; limited number of compounds validated (<10 drugs tested to date)

BeWo, human choriocarcinoma cell line; ECM, extracellular matrix; HBSS, Hanks’ Balanced Salt Solution; hiPSC, human induced pluripotent stem cell; HUVECs, human umbilical vein endothelial cells; JEG-3, human choriocarcinoma cell line; PDMS, polydimethylsiloxane.

**Table 3:** Representative OoC applications for drug transport and safety assessment across the FRT.

Model	Testing Compound/Drug	Key Findings	Mechanism/Readout	Application
Endometrium-on-chip	Levonorgestrel	Dose-dependent increase in barrier permeability; regression of microvasculature observed at higher doses	FITC–dextran permeability assay (Papp); vessel imaging	Screening contraceptive and hormone therapies
	Metabolic substrates and regulators (Glucose, Insulin)	High glucose induced >200 differentially expressed genes and 23 proteins; insulin elicited minimal effects.	RNA-seq; proteomic profiling	Understanding metabolic regulation of endometrial barrier function
Placenta-on-chip	Nutrients (Glucose)	Transfer rates consistent with ex vivo perfusion; CSA-binding infected erythrocytes increased barrier resistance and reduced glucose transport	GLUT1-mediated diffusion (Papp)	Modeling nutrient exchange and infection-modulated transport
	Caffeine	Time-dependent maternal–fetal transfer; fetal exposure substantially lower than maternal levels	LC–MS; fluorescent tracer diffusion	Quantitative assessment of passive permeability and fetal drug exposure
	Paclitaxel, Vancomycin, Glyburide, IgG	Dose-dependent transfer; active efflux (P-gp, BCRP) limited fetal exposure; results consistent with clinical profiles	Transporter inhibition assays; permeability measurements	Evaluating prescription drug safety and transporter-mediated efflux during pregnancy
	Endocrine-disrupting compounds (BPA, PBDE-47/99)	Local trophoblast oxidative stress and inflammatory responses; minimal impact on glucose flux at low dose	Cytokine profiles; ROS assays	Screening reproductive toxicants and oxidative stress response
	Nanoparticles (CuO, TiO_2_, CSA-liposomes)	CuO and TiO_2_ NPs impaired barrier integrity and glucose transport; CSA-liposomes showed increased uptake with syncytialization	ICP–MS; NP transport assays; oxidative stress and viability analysis	Assessing nanotoxicity and designing placental targeted nanotherapies

BCRP, breast cancer resistance protein; BPA, bisphenol A; CSA, chondroitin sulfate A; CuO, copper oxide nanoparticles; FITC, fluorescein isothiocyanate; GLUT1, glucose transporter 1; ICP–MS, inductively coupled plasma mass spectrometry; IgG, immunoglobulin G; LC–MS, liquid chromatography–mass spectrometry; NP, nanoparticle; PBDE, polybrominated diphenyl ether; P-gp, P-glycoprotein; RNA-seq, RNA sequencing; ROS, reactive oxygen species; TiO_2_, titanium dioxide nanoparticles.

## Data Availability

No new data were created or analyzed in this study. Data sharing is not applicable to this article.

## Abbreviations

The following abbreviations are used in this manuscript:

ABC	ATP-binding cassette
AMP	antimicrobial peptide
BCRP	breast cancer resistance protein
BPA	bisphenol A
CFTR	cystic fibrosis transmembrane conductance regulator
CSA	chondroitin sulfate A
CuO	copper oxide
DMEM	Dulbecco’s Modified Eagle Medium
ECM	extracellular matrix
EEC	endometrial epithelial cell
EEO	endometrial epithelial organoid
ENaC	epithelial sodium channel
ESF	endometrial stromal fibroblast
FITC	fluorescein isothiocyanate
FRT	female reproductive tract
GLUT	glucose transporter
HBSS	Hanks’ Balanced Salt Solution
hiPSC	human induced pluripotent stem cell
HPVEC	human placental vascular endothelial cell
HUVEC	human umbilical vein endothelial cell
ICP-MS	inductively coupled plasma mass spectrometry
IgA	immunoglobulin A
IgG	immunoglobulin G
IVPT	in vitro permeation testing
MDR	multidrug resistance
MMP	matrix metalloproteinase
MRP	multidrug resistance-associated protein
MUC	mucin
OATP	organic anion transporting polypeptide
OoC	organ-on-chip
P_app_	apparent permeability coefficient
PBDE	polybrominated diphenyl ether
PDMS	polydimethylsiloxane
PEG	polyethylene glycol
PET	polyethylene terephthalate
ROS	reactive oxygen species
TiO_2_	titanium dioxide
